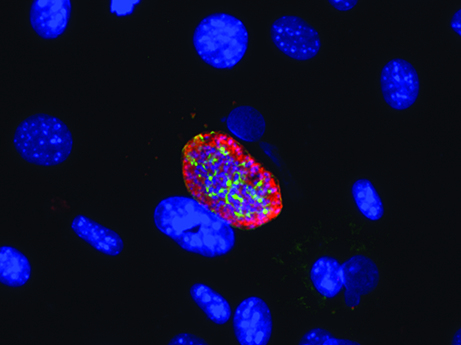# Effects of oxygen on malaria infection: implications for studying liver-stage malaria

**Published:** 2014-02

Malaria is a mosquito-borne infectious disease caused by *Plasmodium*, a type of parasitic protozoan. Worldwide, malaria causes ~1,000,000 deaths per year, with tropical and sub-tropical regions being the most severely affected. Although the primary symptoms and complications associated with malaria are attributable to parasite replication in red blood cells, in the initial stage of infection *Plasmodium* infects and grows in the liver. This liver stage is an attractive target for drug treatment; however, model systems that mimic the normal liver environment and response to parasitic infection are limited. Here, Sangeeta Bhatia and co-authors explored the effects of oxygen concentration on parasitic growth in the liver *in vivo* and *in vitro*. They report that parasitic growth in the livers of infected mice correlates with a natural variation in oxygen levels. Furthermore, exposure of micropatterned co-cultures of primary human hepatocytes and supporting stromal cells to hypoxia enhanced infection efficiency and parasitic development. These findings have important implications for the development of liver-stage malaria platforms for antimalarial drug screening, where oxygen levels should be carefully controlled. Page 215

**Figure:**